# Preclinical evaluation of tolvaptan and salsalate combination therapy in a *Pkd1*-mouse model

**DOI:** 10.3389/fmolb.2023.1058825

**Published:** 2023-01-19

**Authors:** Xuewen Song, Wouter N. Leonhard, Anish A. Kanhai, Gregory R. Steinberg, York Pei, Dorien J. M. Peters

**Affiliations:** ^1^ Division of Nephrology, University Health Network and University of Toronto, Toronto, ON, Canada; ^2^ Department of Human Genetics, Leiden University Medical Center, Leiden, Netherlands; ^3^ Centre for Metabolism, Obesity and Diabetes Research, Department of Medicine, McMaster University, Hamilton, ON, Canada

**Keywords:** salsalate, tolvaptan, combination therapy, preclinical trial, gene expression profiling, ADPKD

## Abstract

**Background:** Autosomal dominant polycystic kidney disease (ADPKD) is the most common genetic disorder and an important cause of end stage renal disease (ESRD). Tolvaptan (a V2R antagonist) is the first disease modifier drug for treatment of ADPKD, but also causes severe polyuria. AMPK activators have been shown to attenuate cystic kidney disease.

**Methods:** In this study, we tested the efficacy of the combined administration of salsalate (a direct AMPK activator) and tolvaptan using clinically relevant doses in an adult-onset conditional *Pkd1* knock-out (KO) mouse model.

**Results:** Compared to untreated *Pkd1* mutant mice, the therapeutic effects of salsalate were similar to that of tolvaptan. The combined treatment tended to be more effective than individual drugs used alone, and was associated with improved kidney survival (*p* < 0.0001) and reduced kidney weight to body weight ratio (*p* < 0.0001), cystic index (*p* < 0.001) and blood urea levels (*p* < 0.001) compared to untreated animals, although the difference between combination and single treatments was not statistically significant. Gene expression profiling and protein expression and phosphorylation analyses support the mild beneficial effects of co-treatment, and showed that tolvaptan and salsalate cooperatively attenuated kidney injury, cell proliferation, cell cycle progression, inflammation and fibrosis, and improving mitochondrial health, and cellular antioxidant response.

**Conclusion:** These data suggest that salsalate-tolvaptan combination, if confirmed in clinical testing, might represent a promising therapeutic strategy in the treatment of ADPKD.

## 1 Introduction

ADPKD is the most common hereditary kidney disease worldwide with an estimated cumulative lifetime risk of ∼1 in 1,000 (Lanktree et al., 2018). Progressive increase in number and size of cysts with age results in the distortion of normal kidney architecture and ultimately, ESRD in a majority of patients (Grantham, 2008). Mutations of two genes, *PKD1* and *PKD2*, encoding for either polycystin-1 or polycystin-2, account for 75–85% and 15–25% of the genetically resolved cases (Peters and Sandkuijl, 1992; Cornec-Le Gall et al., 2013; Heyer et al., 2016; Hwang et al., 2016). Dysregulation of various cellular pathways has been implicated in ADPKD. Reduced polycystin function is thought to cause the increased cAMP signaling, which is a key driving mechanism for cyst growth and fluid secretion. The mTORC1 pathway regulates cell growth and proliferation, and is hyperactivated in cystic lesions and participates in in modulating cyst growth (Harris and Torres, 2014; Bergmann et al., 2018). Both cAMP and mTORC1 have been experimentally validated to be important therapeutic targets (Grantham, 2003; Torres et al., 2004; Shillingford et al., 2006; Shillingford et al., 2010) and clinically tested (Serra et al., 2010; Walz et al., 2010; Novalic et al., 2012; Torres et al., 2012; Torres et al., 2017). Tolvaptan, an oral V2 vasopressin receptor (V2R) antagonist which lowers [cAMP]_i_ in cystic kidney tissues has been recently shown to delay progression of ADPKD in two large randomized clinical trials (Torres et al., 2012; Torres et al., 2017) and is now approved for clinical use in multiple countries. However, severe polyuria (typically 6–10 L/day) from tolvaptan-induced nephrogenic diabetes insipidus (NDI) is a common side-effect that negatively impacts patient quality of life (Torres et al., 2012). For pharmacological inhibition of mTORC1, high dose treatment was highly effective in experimental studies of ADPKD (Shillingford et al., 2006; Shillingford et al., 2010; Novalic et al., 2012), but low dose treatment did not significantly improve the cystic phenotype in mice (Novalic et al., 2012). Concomitantly, clinical trials of mTORC1 inhibitors showed a lack of efficacy with low-dose treatment (Serra et al., 2010) and poor tolerability with high-dose treatment (Walz et al., 2010). Notably, mTORC1 and AMPK are two diametrically opposing sensors of energy metabolism, which regulate cell growth and proliferation (Hardie, 2013; Garcia and Shaw, 2017; Herzig and Shaw, 2018; Steinberg and Carling, 2019; Song et al., 2020). Accumulating evidence suggests that therapeutic AMPK activation (e.g., metformin, 2-deoxyglucose or dietary interventions) attenuates cystic kidney disease severity by inhibiting mTORC1, CFTR, inflammation/fibrosis, and restoring mitochondrial energy metabolism in *Pkd1* mutant animal models (Takiar et al., 2011; Chiaravalli et al., 2016; Kipp et al., 2016; Riwanto et al., 2016; Warner et al., 2016; Leonhard et al., 2019; Lian et al., 2019; Torres et al., 2019; Pastor-Soler et al., 2022).

Salsalate is a prodrug dimer of salicylate, which activates AMPK through a direct interaction with the Ser108 residue of the AMPK β1 isoform (Hawley et al., 2012). Independent of its AMPK effects and at clinical concentrations, it increases mitochondrial proton conductance to uncouple oxidative phosphorylation (OXPHOS) *in-vitro* (Smith et al., 2016). Salsalate also reduces inflammation through a number of distinct mechanisms including activation of AMPK. Compared to aspirin (acetyl-salicylate), salsalate displays weak cyclooxygenase enzyme inhibitory activity at its usual dose and is associated with minimal bleeding risk and fewer GI side-effects (Anderson et al., 2014). We have recently tested the efficacy of salsalate in an adult-onset *Pkd1* conditional KO mouse model, and found that at clinically relevant doses it effectively attenuated cystic disease severity (Leonhard et al., 2019). The purpose of the present study was to evaluate whether the combined use of tolvaptan with salsalate inhibited PKD with increased therapeutic efficacy and the potential mechanisms mediating these effects.

## 2 Materials and methods

### 2.1 Mouse experimental protocol

**FIGURE 1:**
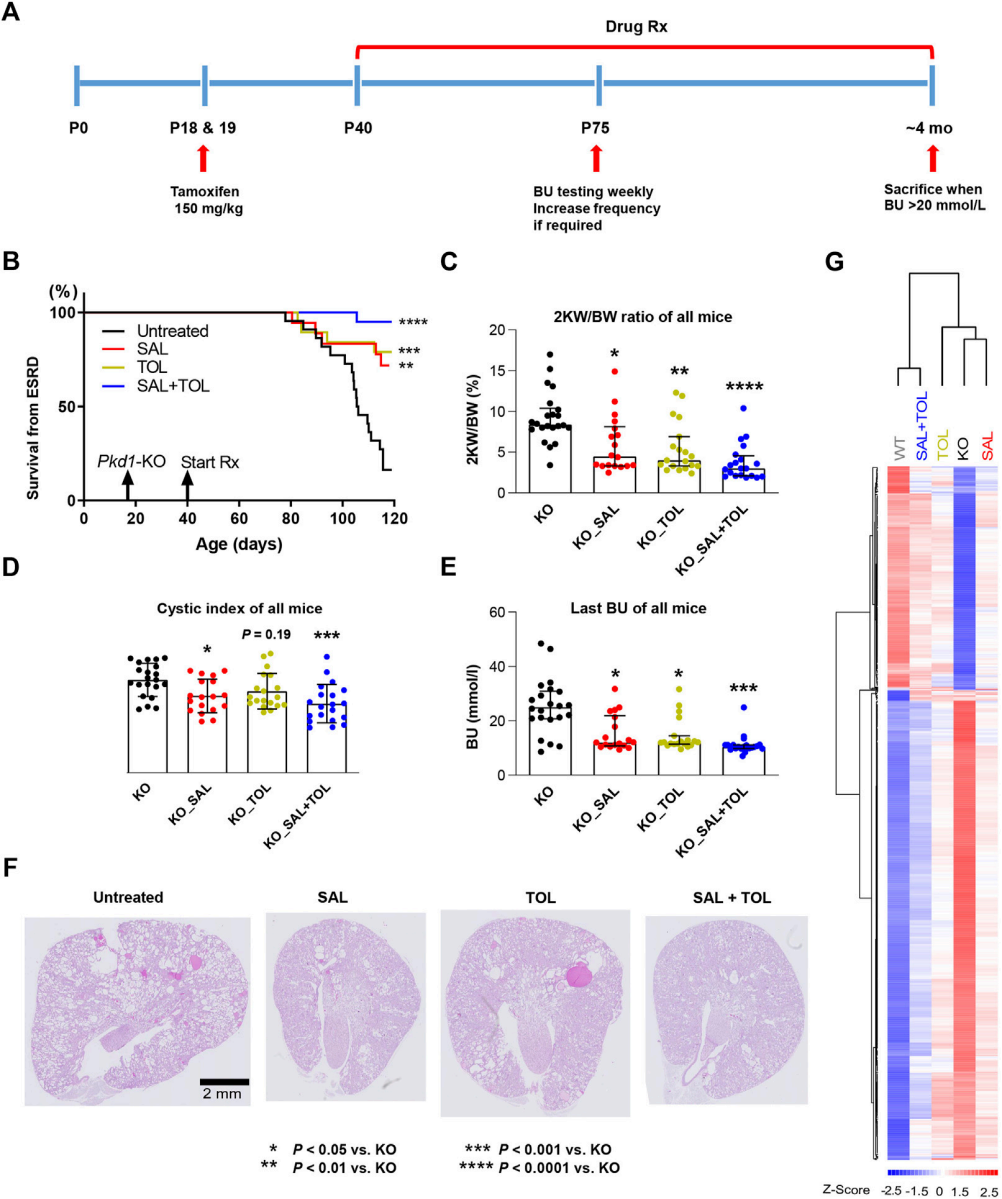
Combined treatment improved kidney survival and cystic parameters. **(A)** Experimental protocol. We tested the efficacy of salsalate (SAL), tolvaptan (TOL) or combination (SAL + TOL) in a tamoxifen-inducible *Pkd1* conditional KO mouse model (iKsp-*Pkd1* ^del^ mice). Male mice were treated with tamoxifen at days P18 and P19 to inactivate *Pkd1* gene and drug treatment began on day P40. Untreated mutant mice began to develop advanced kidney failure at around ∼ P105. BU monitoring *via* tail-vein blood sampling began at P75 to identify those mice with kidney failure; all mice with a BU > 20 mmol/L were considered to have ESRD and sacrificed. When ∼75% of the untreated mutant mice reached ESRD, all the mice from all study groups were sacrificed. **(B)** Kidney survival of untreated *versus* treated mice. **(C)** 2 KW/BW%, **(D)** cystic indices, and **(E)** BU of all mice at their respective endpoints. **(F)** Representative PAS-stained kidney sections (i.e., the median) from different study groups. All *Pkd1* mutant mice with and without ESRD were included in **(B–E)**; untreated KO (n = 22), SAL (n = 18), TOL (n = 19), SAL + TOL (n = 20). **(G)** Hierarchical clustering using top 1,000 differentially expressed genes between KO (n = 12) and WT (n = 10) kidneys showing the SAL (n = 11), TOL (n = 12), and SAL + TOL (n = 12) treatments attenuated gene expression changes in *Pkd1* mutant kidneys, but co-treatment showed overall larger fold changes than the monotherapies. The row and column dendrograms display the distance or similarity between genes and samples, respectively. Columns represent five groups (WT, KO, KO_SAL, KO_TOL, and KO_SAL + TOL) and rows represent the Z-score scaled average gene expression levels of each group. White is the mean expression across all groups (set to 0), red indicates greater than the mean and blue, less than the mean. Z-scores are computed on a gene-by-gene basis by subtracting the mean and then dividing by the standard deviation.

The experimental design of our study is shown in Figure 1A. All the study groups were run concurrently in one large experiment. We used the iKsp-*Pkd1* ^del^ inducible conditional KO mice on a C57BL6/J congenic background for the testing (Lantinga-van Leeuwen et al., 2007). Because of a gender dimorphism in this model with male mice displaying more severe disease, we studied only male mice to minimize disease variability. Detailed mouse experimental protocol has been described previously (Leonhard et al., 2019; Kanhai et al., 2020). Kidney-specific deletion of the *Pkd1* gene was induced with tamoxifen (150 mg/kg, by oral gavage) at days P18 and P19. After weaning, mice treated with tamoxifen were randomized into four groups: untreated (KO, n = 22), salsalate (KO_SAL, n = 18), tolvaptan-SD (KO_TOL, n = 19), or combination of tolvaptan-SD and salsalate (KO_SAL + TOL, n = 20) (Supplementary Figure S1). Drug treatment began on day P40. Drug doses for salsalate and tolvaptan have been established in our previous studies (Leonhard et al., 2019; Kanhai et al., 2020). Treatment groups received food pellets, supplemented with either 0.25% salsalate (AK-Scientific, #F817, resulting in a dose of 400 mg/kg/day, previously shown to result in median serum salicylate levels of 222 (IQR: 108–372) μM) (Leonhard et al., 2019) and/or 0.15% tolvaptan-SD (66.7% tolvaptan), which is the equivalent of the commonly used dose in preclinical research of 0.1% tolvaptan (Kanhai et al., 2020). Tolvaptan-SD is a tolvaptan formulation designed to improve the drug’s oral bioavailability (kindly provided by Otsuka Pharmaceuticals, Tokyo, Japan). Untreated control mice received food pellets generated by the same protocol, but without any drug. To monitor kidney function, blood urea (BU) was measured weekly starting at day P75 using blood samples from the tail vein (Reflotron technology, Roche). Mice with a BU > 20 mmol/L were considered to have ESRD and sacrificed. When ∼75% of the untreated mice reached ESRD at 111–120 days of age, all mice from the control and other treatment groups were sacrificed. For mice with BU > 20 mmol/L, the time to ESRD was calculated by linear interpolation of the ages between the last two BU measurements. The age at ESRD or at censoring (i.e., mice without ESRD at the time of their last BU measurement between 111 and 120 days of age) was used for Kaplan Meier survival analysis. Kidney disease severity was assessed using the ratio of two-kidney weight/body weight (2 KW/BW%) and cystic index. ‘Wild-type’ control mice are iKps-*Pkd1* ^*lox*^ mice without tamoxifen treatment, sacrificed at 144 days of age. All animal procedures were approved by the Animal Experiment Committee of the Leiden University Medical Center and the Commission Biotechnology in Animals of the Dutch Ministry of Agriculture, and performed in accordance to Directive 2010/63/EU for animal experiments.

### 2.2 Histology

Kidneys were fixed overnight in 4% buffered formaldehyde solution and embedded in paraffin. Kidney sections (4 μm) were stained with Periodic acid-Schiff (PAS) using standard protocols. Cystic indices were determined based on the established scoring system using ImageJ software (public domain software; National Institutes of Health) and defined as the percentage of total cystic area relative to total kidney area (Leonhard et al., 2016; Kanhai et al., 2020).

### 2.3 Gene expression and bioinformatics analysis

A flow-chart for the bioinformatics analysis is outlined in Supplementary Figure S2A. Using the Mouse Gene 2.0 ST Arrays (Affymetrix), we performed global gene profiling on 57 kidney samples including wild type (WT, n = 10), KO (n = 12), KO_SAL (n = 11), KO_TOL (n = 12), and KO_SAL + TOL (n = 12). The mutant kidneys were selected around the median of 2 KW/BW% from each group. Microarray experiments were performed by the Centre for Applied Genomics Core at the Hospital for Sick Children (Toronto, Ontario, Canada). Data processing, quality measures, normalization, and statistical analyses for differentially expressed genes were performed using the Affymetrix Transcriptome Analysis Console (TAC 4.0) with a false discovery rate (FDR) *p*-value ≤5%. To get a list of genes for gene set enrichment analysis (GSEA), probes without gene symbols or with very low expression levels (mean expression values across all samples <30) were filtered out prior to analysis. We used Enrichr as the primary tool for GSEA (Kuleshov et al., 2016). In short, differentially expressed genes from each comparison were split into two groups of up-regulated and down-regulated genes. The lists of up-regulated and down-regulated genes were each used for GSEA. In this study, we used Reactome_2016 (database of biological pathways, n = 1,530) for GSEA. Reactome pathway database uses a hierarchical organization that represents broad biological concepts at the uppermost hierarchical level, which are divided at the next hierarchical level into sub-pathways and sub-sub pathways (Jassal et al., 2020). The GSEA outputs from Reactome pathway analysis include all levels of the pathway hierarchy. Enrichr computes two key statistics for the report: 1) The adjusted *p*-value is computed using Fisher’s exact test and adjusted using the Benjamini–Hochberg method. 2) Odds ratio is computed to measure the strength of association. The combined score is a multiplication of the odds ratio by the negative natural log of the adjusted *p*-value. It provides a balance between these two methods of ranking. We defined enriched gene sets by an adjusted *p*-value ≤0.01 and combined score ≥10.

Hierarchical clustering analysis and heatmap were performed using the DNA-Chip Analyzer (dChip) software package (Li and Hung Wong, 2001). In the heatmap, each column represents an individual sample; each row represents the Z-score scaled gene expression levels across all samples (Z-scores are computed on a gene-by-gene basis by subtracting the mean and then dividing by the standard deviation, and reflect the distance from the mean in units of standard deviation). White is the mean Z-score (set to 0), red indicates greater than the mean and blue, less than the mean. *Pkd1* mutant kidneys in the heatmap were ordered by 2 KW/BW%.

### 2.4 DNA and RNA isolations and quantitative PCR (qPCR)

Total DNA containing mitochondrial and nuclear DNA (nDNA) was isolated by using QIAamp DNA Mini Kit (Qiagen). Total RNA was isolated by using miRNeasy Mini Kit (Qiagen) with an on-column DNA digestion step to minimize genomic DNA contamination. cDNA was generated using High-Capacity cDNA Reverse Transcription Kit (Applied Biosystems). Real time qPCR was carried out on a ViiA 7 Real-Time PCR System (Applied Biosystems), using Power SYBR® Green PCR Master Mix (Applied Biosystems). Mitochondrial DNA (mtDNA) copy numbers were estimated by the ratio of mitochondrial gene *16S rRNA* and nuclear gene *B2m*. mRNA expression levels of *Ccl2* and *Ppacgc1a* were normalized to *Hprt1* expression and expressed as relative fold change (FC) over WT kidneys. Primer sequences of target genes used in qPCR are listed in Supplementary Table S1.

### 2.5 Protein extraction and western blot analysis

The mutant kidneys were selected around the median of 2 KW/BW% from each group. Snap-frozen kidney tissue samples were homogenized in 4°C lysis buffer containing 50 mM HEPES pH 7.4, 150 mM NaCl, 100 mM NaF, 10 mM Na pyrophosphate, 5 mM EDTA, 250 mM sucrose, 1 mM DTT, and 1 mM Na-orthovanadate, 1% Triton X, 0.2% SDS and Complete protease inhibitor cocktail (Cat#11836153001, Roche Diagnostics). The lysates were incubated for 30 min at 4°C and then centrifuged for 15 min at 13,000 rpm at 4°C. Supernatants were collected, aliquoted and stored at −80°C. Protein concentrations were determined using Protein Assay Kit (Bio-Rad). Proteins were denatured in Laemmli sample buffer, separated on Criterion™ TGX™ Precast gradient gels (Bio-Rad) and then transferred onto PVDF membranes (Bio-Rad) by using Trans-Blot Turbo System (Bio-Rad). The membranes were blocked in blocking buffer (150 mM NaCl, 20 mM Tris, 5% skim milk, 0.1% Tween 20) for 1 h, and then incubated with primary antibodies overnight at 4°C or 1.5 h at room temperature according to the manufacturers’ instructions. Subsequently, primary antibody binding was detected with horseradish peroxidase (HRP)-conjugated anti-rabbit, or anti-mouse secondary antibodies, and proteins were visualized with Clarity™ or Clarity™ Max Western ECL Substrate (Bio-Rad) using a ChemiDoc XRS + Gel Imaging System (Bio-Rad). Relative expression of target protein was normalized with GAPDH expression using Image Lab™ software (Bio-Rad). For determination of phospho/total protein levels, immunoblots were first probed for phospho levels, then stripped at RT for 30 min using Re-Blot Plus Mild Antibody Stripping Solution (EMD Millipore) and re-probed overnight to detect total protein levels. The primary antibodies for total S6K1 (Cat#9202, RRID:AB_331676), p-Thr389 S6K1 (Cat#9234, RRID:AB_2269803), total SRC (Cat#2123, RRID:AB_2106047), p-Ser17 SRC(Cat#5473, RRID:AB_10829921), total NFκB p65 (Cat#8242, RRID:AB_10859369), p-Ser536 NFκB p65 (Cat#3033, RRID:AB_331284), total ERK1/2 (Cat#4695, RRID:AB_390779), p-Thr202/Tyr204 ERK1/2 (Cat#4370, RRID:AB_2315112), PCNA (Cat#2586, RRID:AB_2160343), cGAS (Cat#31659, RRID:AB_2799008), STING (Cat#13647, RRID:AB_2732796), GAPDH (Cat#5174, RRID:AB_10622025) were obtained from Cell Signaling Technology. The antibodies for PGC1α (Cat#sc-517380, RRID:AB_2755043) and CDK2 (Cat#sc-6248, RRID:AB_627238) were from Santa Cruz Biotechnology, and the antibodies for TNF-α (Cat#60291-1-Ig, RRID:AB_2833255), α-SMA (Cat#14395-1-AP, RRID:AB_2223009), TFAM (Cat#22586-1-AP, RRID:AB_11182588), NRF1 (Cat#12482-1-AP, RRID:AB_2282876) were from Proteintech. The secondary antibodies for HRP-conjugated anti-rabbit antibody (Cat#7074, RRID:AB_2099233) and HRP-conjugated anti-mouse antibody (Cat#7076, RRID:AB_330924) were obtained from Cell Signaling Technology.

### 2.6 Statistical analysis

Statistical analysis was performed using GraphPad Prism 9 software (San Diego, CA, United States). All results are expressed as dot-blots with median and interquartile ranges for non-normally distributed data or mean ± SD for normally distributed data. Comparisons involving more than two groups were performed by Kruskal–Wallis non-parametric test or one-way ANOVA followed by Dunnett’s or Tukey’s *post hoc* test; *p*-values corrected for multiple comparisons were reported. The Log Rank (Mantel-Cox) test was used for the Kaplan Meier survival analyses. A two-tailed chi-square test with Yates’ correction was performed to determine whether combined drug treatment statistically altered more genes than single treatments.

## 3 Results

### 3.1 Combined salsalate and tolvaptan treatment improved kidney survival and cystic parameters

The effect of the treatments on the ADPKD phenotype was evaluated by analyzing kidney survival, 2 KW/BW%, cystic index, and BU of all mice at their respective endpoints. Consistent with previous studies (Leonhard et al., 2019; Kanhai et al., 2020), both salsalate and tolvaptan monotherapy were effective in improving kidney survival (Figure 1B), reducing 2 KW/BW% (Figure 1C), cystic index (Figure 1D), and BU levels (Figure 1E), as compared to untreated *Pkd1* mutant mice. The combined treatment reached a higher level of statistical significance on the same parameters. Compared to untreated mutant mice, the combined treatment strongly improved kidney survival (*p* < 0.0001), reduced 2 KW/BW% (*p* < 0.0001), cystic index (*p* < 0.001) and BU (*p* < 0.001) (Figures 1B–E), although the comparisons between monotherapy and combination treatment were not significantly different. In addition, treatment with salsalate, tolvaptan or in combination was associated with milder cystic disease histology compared to untreated animals (Figure 1F). These data indicate that the therapeutic effect of salsalate was similar to that of tolvaptan and suggest that the combined treatment might be more effective than either agent as monotherapy.

There is some evidence of reduced body weight in salsalate-treated animals despite similar energy intakes in the context of diabetes and metabolic dysfunction (Cao et al., 2011; Nie et al., 2017). However, we found body weight of the mice did not differ between the different groups, as reported previously (Leonhard et al., 2019).

### 3.2 The transcriptional profiles of drug combination reflected the phenotypic characteristics

To examine the potential mechanisms mediating these effects, we performed global gene expression profiling on 57 kidney samples (Supplementary Figure S2A). Principal component analysis (PCA) of the gene expression data clustered apart kidney samples from different genotypes, untreated and treated groups. However, PCA did not give a clear distinction between groups with different treatments (Supplementary Figure S2B). We then performed the clustering analysis to partition genes and samples with similar trends. The heatmap displayed the gene expression patterns of top 1,000 differentially expressed genes between WT and mutant kidneys, and all these changes were attenuated by the treatments. Importantly, salsalate and tolvaptan treatment groups displayed similar transcriptional profiles, but the co-treatment group showed overall larger magnitudes of fold changes than the monotherapies, supporting the phenotypic data described above (Figure 1G; Supplementary Figure S3).

To delineate the pathways associated with PKD progression and treatment effects, we performed gene and pathway analysis of WT, salsalate, tolvaptan, and salsalate plus tolvaptan treated kidneys vs. the untreated KO controls (i.e., WT vs. KO, SAL vs. KO, TOL vs. KO, and SAL + TOL vs. KO), the results were summarized in Supplementary Table S2. Compared to untreated KO kidneys, the combined treatment significantly altered more genes than the monotherapies (two-tailed chi-square test with Yates’ correction, *p* < 0.0001). At FDR *p* ≤ 0.05 with different fold change thresholds (i.e., ≥ 1.5, ≥2, and ≥3), the numbers of genes down-regulated by the combined treatment were greater than the theoretical sum of down-regulated genes by salsalate and tolvaptan treatment alone (Supplementary Table S2). To explore and compare the pathways associated with PKD progression and treatment effects, we merged and sorted the dysregulated pathways from four comparisons by different hierarchical levels (Supplementary Tables S3, S4). Consistent with our previous findings in human *PKD1* renal cysts and mouse studies (Song et al., 2009; Leonhard et al., 2019; Malas et al., 2020), we found that *Pkd1* mouse mutant kidneys displayed a rich network of up-regulated signaling pathways for protein translation, cell cycle progression, growth factors/receptors, G-protein-coupled receptors (GPCR), Rho GTPase, RAS/RAF/MAPK, as well as innate immunity and fibrosis (Supplementary Table S3). On the other hand, the majority of the down-regulated pathways in *Pkd1* mutant kidneys represent metabolic pathways (Supplementary Table S4). Most of these pathways were significantly attenuated by the treatments. Combined treatment reached a higher level of statistical significance on some pathways, such as protein translation, cell cycle progression, signaling by Rho GTPases, apoptosis, senescence, Hippo signaling, as well as mitochondrial protein import, translation, and biogenesis.

To attempt to identify genes that were additively, or synergistically altered by the combination therapy, we looked for differentially expressed genes between combination therapy and monotherapies (i.e., SAL + TOL vs. SAL, and SAL + TOL vs. TOL), and found 404 common genes from these two comparisons (Supplementary Table S2). However, all these genes tended to strongly correlate with PKD progression. Given mild phenotypic difference between the mice receiving combination therapy and monotherapies, the observed gene expression changes likely reflect phenotypic differences between these groups, rather than treatment related alterations.

We also attempted to identify genes specifically altered by either tolvaptan or salsalate (i.e., SAL vs. TOL). Compared to tolvaptan treatment alone, salsalate treatment resulted in 84 dysregulated genes (Supplementary Table S2). Among the genes of interest, increased mRNA levels of 11 lipid metabolism genes were found in salsalate treated kidneys. On the other hand, Prlr, and class V HLH proteins Id1-4 displayed higher mRNA levels in tolvaptan treated kidneys. However, a majority of these genes either displayed mild fold changes, or were with low expression levels, and more experiments are needed to reach conclusions.

### 3.3 Combined salsalate and tolvaptan treatment attenuated key pathogenic mediators in *Pkd1* mutant mouse kidneys

Aberrant mTORC1 activation and increased cAMP signaling in cystic tissues are two key pathogenic mechanisms driving cyst growth in ADPKD (Harris and Torres, 2014; Bergmann et al., 2018). mTORC1 regulation of protein synthesis is primarily mediated by phosphorylation of p70S6 kinase 1 (S6K1) and eIF4E binding protein (4EBP1), which drives mRNA translation initiation and 4EBP1 dissociation from eIF4E allowing for 5’cap-dependent mRNA translation, respectively (Holz et al., 2005; Thoreen et al., 2012). By transcriptome-based pathway analysis, multiple gene sets for protein translation were up-regulated in *Pkd1* mutant kidneys (vs. WT). Specifically, among the sub-pathways under translation, the cap-dependent translation initiation was highly up-regulated in *Pkd1* mutant kidneys (Adjusted *p* = 1.58E-11), but both salsalate and tolvaptan monotherapy only displayed a trend in attenuating this pathway (salsalate, Adjusted *p* = 0.015; tolvaptan, Adjusted *p* = 0.19) associated with the reduction of mRNA expression of 32 and 26 genes, respectively. However, the combined treatment induced the reduction reached a statistical significance (Adjusted *p* = 6.04E-05), with more down-regulated genes in this pathway (n = 47) (Supplementary Table S3).

Salicylate has been shown to exert anti-proliferative effects through inhibiting cell cycle regulatory proteins cyclin A2 and CDK2 (Dachineni et al., 2016). As expected, we found that salsalate alone or in combination, but not tolvaptan, significantly attenuated cell cycle pathway associated with a reduction of mRNA expression of 145, 221, and 91 genes, respectively (salsalate: Adjusted *p* = 2.50E-06; tolvaptan: Adjusted *p* = 1; salsalate + tolvaptan: Adjusted *p* = 4.15E-18). The combined treatment reached a higher level of statistical significance associated with more down-regulated genes than the monotherapies (Supplementary Table S3). Although we found no definitive enrichment of cell cycle pathway in tolvaptan treated kidneys, a number of genes in cell cycle pathway (n = 91), such as *Cdk2*/*4*/*6*, *Ccnd2*, and *Mcm4*/*5*, were indeed down-regulated upon tolvaptan treatment (Supplementary Table S3).

In ADPKD, cAMP/PKA dependent Ras/Raf/MEK/ERK signaling induces cell proliferation and cyst growth (Yamaguchi et al., 2003). We found, salsalate, tolvaptan, or in combination significantly attenuated RAS, RAF, ERK pathways, but the combined treatment induced the reduction on larger numbers of genes than the individual drugs used alone (Supplementary Table S3).

**FIGURE 2:**
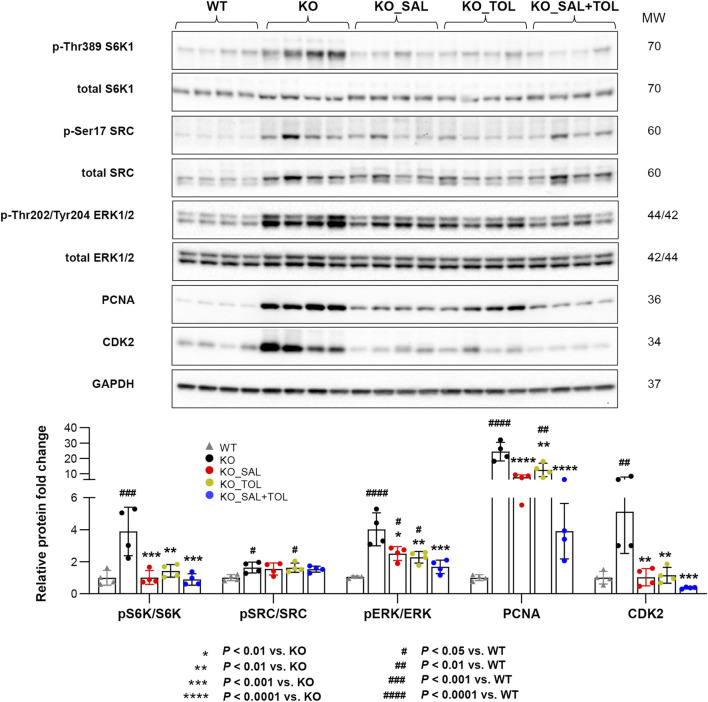
The combined treatment attenuated expression of pathogenic mediators in *Pkd1* mutant mouse kidneys. The phosphorylation levels of S6K1, SRC, and ERK1/ERK2, as well as the total protein levels of PCNA and CDK2 in WT, untreated and treated mutant mouse kidneys by Western blotting. GAPDH was used as a loading control. The mutant kidneys were selected around the median of 2 KW/BW% from each group.

To validate our microarray analysis, we performed western blot for the key proteins and phosphorylated proteins involved in these pathways. We found that *Pkd1* mutant mouse kidneys (vs WT) displayed increased expression of protein markers for mTORC1 activity (i.e., levels of p-Thr389 S6K1), PKA activity (i.e., levels of p-Ser17 SRC, PKA is the main downstream target of cAMP in cystogenesis), cell proliferation (i.e., p-Thr202/Tyr204-ERK1/2, PCNA), and cell cycle progression (i.e., CDK2). All of these changes (except p-SRC/SRC levels) were significantly attenuated by all treatments, and the combined treatment tended to reduce the levels of p-S6K1/S6K1 ratio, p-ERK/ERK, PCNA and CDK2 as compared to mice treated with salsalate or tolvaptan alone, although the difference did not reach statistical significance (Figure 2).

### 3.4 Combined salsalate and tolvaptan treatment attenuated kidney injury, inflammation and fibrosis

**FIGURE 3:**
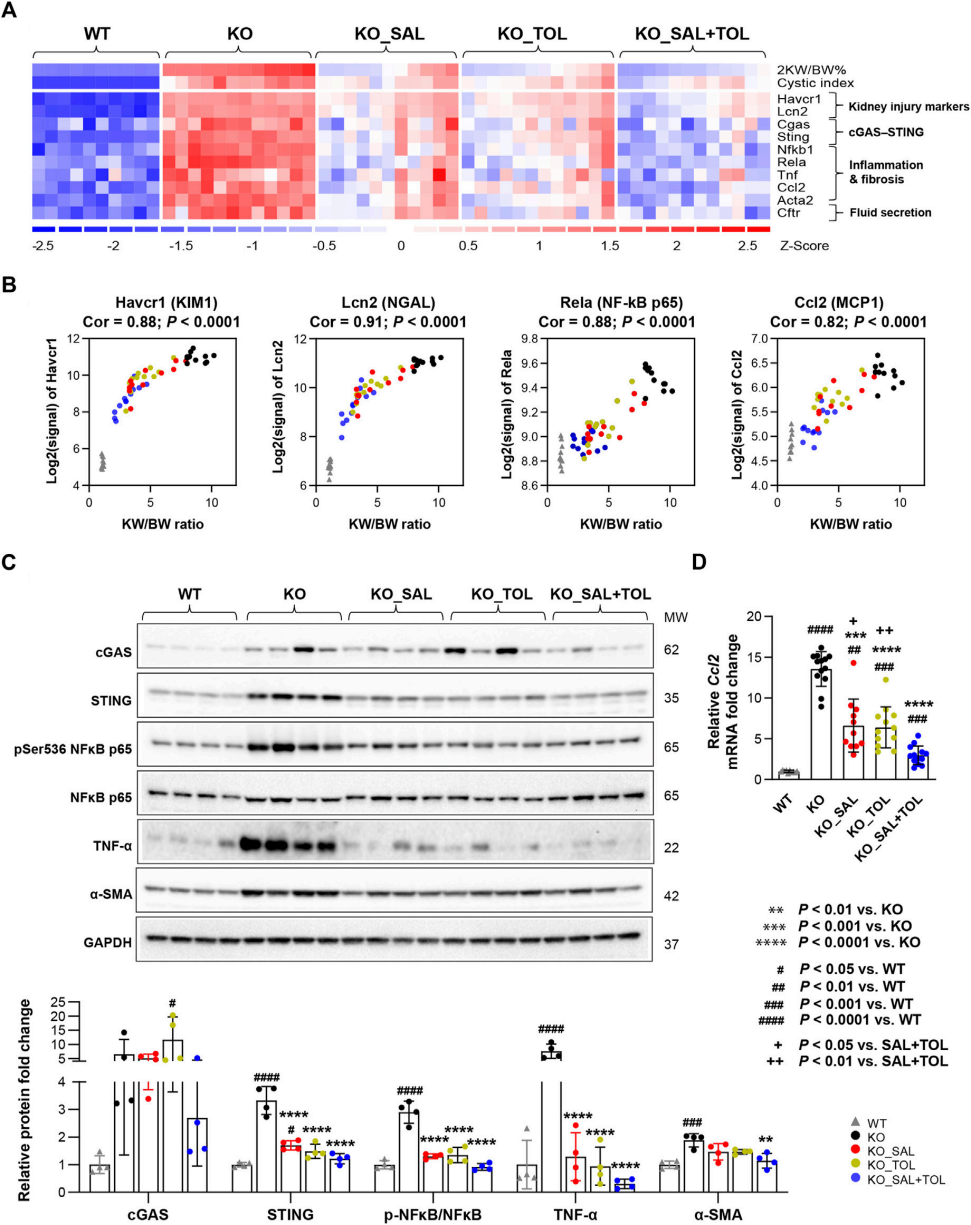
The combined treatment attenuated expression of kidney injury, inflammation, and fibrosis markers in *Pkd1* mutant mouse kidneys. **(A)** Heatmap showed the increased mRNA expression for kidney injury markers (*Havcr1*, *Lcn2*), components of cytosolic DNA sensor (*Cgas*, *Sting*), inflammatory transcription factor NFκB subunits (*Nfkb1*, *Rela*), inflammatory cytokines (*Tnf*, *Ccl2*), fibrosis marker (*Acta2*), as well as chloride channel (*Cftr*) in *Pkd1* mutant kidneys; all these changes were attenuated by the treatments. **(B)** Transcript levels (microarray) of *Havcr1*, *Lcn2*, *Rela*, and *Ccl2* correlated with 2 KW/BW% in *Pkd1* mutant kidneys. **(C)** Relative protein levels of cGAS, STING, NFκB activity (pSer536 NF-kB p65), TNF-α, and α-SMA (Acta2) in WT, untreated and treated mutant mouse kidneys by Western blot. GAPDH was used as a loading control. **(D)** Relative mRNA levels of *Ccl2* in WT, untreated and treated mutant mouse kidneys by RT-qPCR.

Kidney injury, inflammation, and fibrosis are common findings in ADPKD (Weimbs, 2007; Song et al., 2017; Formica and Peters, 2020; Zimmerman et al., 2020). Aberrant kidney injury-repair process plays a role in the progression of ADPKD and we previously reported an injury-repair profile in *Pkd1*-mutant kidneys (Malas et al., 2017; Formica and Peters, 2020). *Havcr1* and *Lcn2,* encoding tubular injury markers Kim1 and NGAL, were identified as top one and three induced genes in *Pkd1* mutant kidneys (vs WT) by 54-fold and 19-fold, respectively. Salsalate, tolvaptan or in combination resulted in the reduction of their transcript levels; but co-treatment showed larger fold changes than the monotherapies (Figure 3A). In addition, transcript levels (microarray) of *Havcr1* and *Lcn2* positively correlated with 2 KW/BW% in *Pkd1* mutant kidneys (Figure 3B). Tissue injury can initiate an inflammatory response through the actions of damage-associated molecule patterns (DAMPs), which can trigger innate immunity by interacting with pattern recognition receptors (Anders and Schaefer, 2014). We found gene sets associated with activation of innate immunity, and extracellular cellular matrix (ECM) accumulation/remodeling in *Pkd1* mutant kidneys, but were significantly attenuated by all three treatments (Supplementary Table S3). The heatmap displayed the increased gene expression of important components of the innate immune system, and the markers for fibrosis in *Pkd1* mutant kidneys (vs. WT), which were all attenuated by all treatments (Figure 3A; Supplementary Figure S4). Concurrently, transcript levels (microarray) of p65 (*Rela*) subunit of inflammatory transcription factor NF-kB, and its target gene *Ccl2*, also positively correlated with PKD disease severity (Figure 3B). Western blot analysis showed the activation of NFκB (pSer536 NF-kB p65), and increased protein expression of inflammatory cytokine TNF-α and fibrosis marker α-SMA in *Pkd1* mutant kidneys. Most of these changes were significantly attenuated by the three treatments (Figure 3C). qRT-PCR analysis confirmed the *Ccl2* gene expression result from microarray analysis. Notably, the combined treatment significantly decreased *Ccl2* mRNA levels as compared to the monotherapies (Figure 3D). Mitochondrial DAMPs (e.g., mtDNA, ATP, and TFAM) have been identified as important mediators of the innate immune response. Cytosolic mtDNA released from damaged mitochondria can be detected by intracellular DNA sensors, and subsequently trigger downstream innate immune response (Grazioli and Pugin, 2018; Riley and Tait, 2020). Importantly, *Sting*, encoding stimulator of interferon genes, a key adaptor protein for cytosolic DNA sensors, was highly induced in *Pkd1* mutant kidneys by 5.1-fold. Accordingly, multiple cytosolic DNA sensors (*Cgas*, *Tlr9*, *Zbp1*, and *Aim2*) were also moderately induced in *Pkd1* mutant kidneys; all of these changes were attenuated by all treatments, but co-treatment showed overall larger fold changes than the monotherapies (Figure 3A; Supplementary Figure S4). By Western blot analysis, we found the protein expression levels of STING were consistent with its mRNA levels. However, the protein level of cGAS was not statistically significant difference between groups, although it is obvious that the results looks different between WT and KO groups. This is probably due to large within-group variation, low sample numbers and multiple experimental conditions tested (Figure 3C). Collectively, these data suggest that cytosolic mtDNA and other DAMPs derived from damaged cells may play important role in triggering activation of innate immunity in *Pkd1* mutant kidneys.

### 3.5 Combined salsalate and tolvaptan treatment improved gene expression of molecular pathways related to oxidative metabolism by modulating mitochondrial biogenesis, antioxidant response, and mitophagy

**FIGURE 4:**
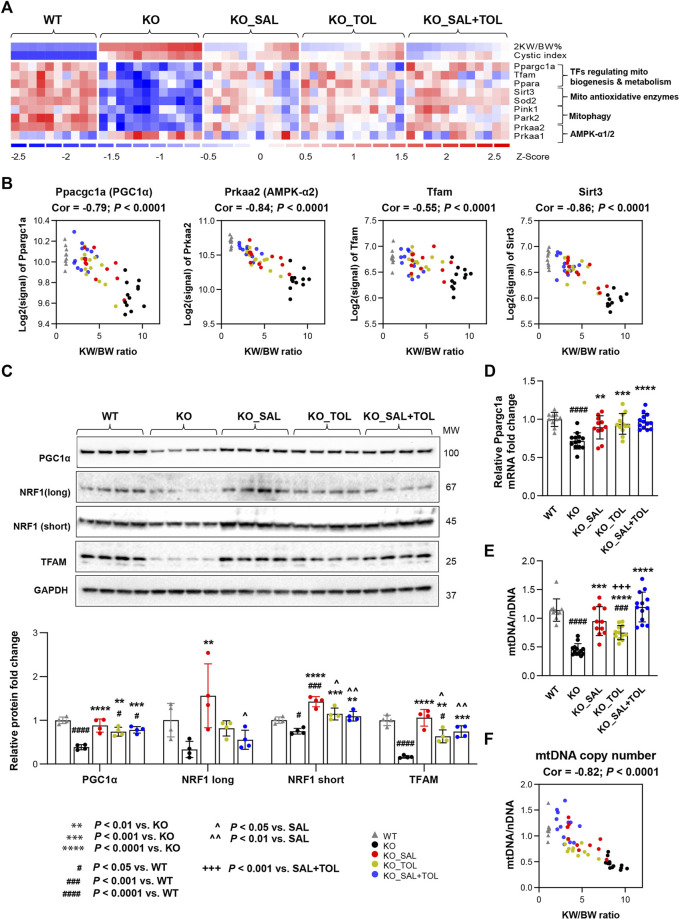
The combined treatment improved expression of components of metabolic pathways and mitochondrial DNA copy numbers in *Pkd1* mutant kidneys. **(A)** Heatmap showed the decreased mRNA expression for key genes in mitochondrial biogenesis, metabolism, antioxidant, and mitophagy in *Pkd1* mutant kidneys; all these changes were attenuated by treatments. **(B)** Transcript levels (microarray) of *Ppargc1a*, *Prkaa2*, *Tfam* and *Sirt3* inversely correlated with 2 KW/BW% in *Pkd1* mutant kidneys. **(C)** Relative protein levels of PGC1α, NRF1 and TFAM in WT, untreated and treated mutant mouse kidneys by Western blot. GAPDH was used as a loading control. **(D)** Relative mRNA levels of *Ppargc1a* in WT, untreated and treated mutant mouse kidneys by RT-qPCR. **(E)** Relative mitochondrial DNA copy numbers in WT, untreated and treated mutant mouse kidneys by qPCR. **(F)** An inverse correlation between mtDNA/nDNA and 2 KW/BW% in *Pkd1* mutant kidneys.

Mitochondrial dysfunction contributes to the pathogenesis of acute kidney injury, abnormal kidney repair and chronic kidney disease. Multiple quality control mechanisms act coordinately to maintain mitochondrial homeostasis (Tang et al., 2021). In *Pkd1* mutant kidneys (vs. WT), we confirmed global depression of mitochondrial energy metabolism associated with down-regulation of pyruvate metabolism, the citric acid (TCA) cycle, OXPHOS, fatty acid oxidation (FAO), branched-chain amino acid catabolism, mitochondrial protein translation and import, and Pink/Parkin mediated mitophagy (Song et al., 2009; Menezes et al., 2016; Malas et al., 2017; Padovano et al., 2018; Podrini et al., 2019; Malas et al., 2020). Metabolism of amino acids, lipids/lipoproteins, vitamins and detoxification of reactive oxygen species (ROS) were also highly suppressed. All these changes were attenuated by all treatments (Supplementary Table S4; Figure 4A; Supplementary Figure S5). OXPHOS system (respiratory electron transport and ATP synthesis) was identified as one of the top down-regulated pathways, and associated with decreased mRNA expression of 94 out of 104 pathway genes in *Pkd1* mutant kidneys. Salsalate, tolvaptan, alone or in combination, significantly improved the OXPHOS system associated with an induction of mRNA expression of these genes (Supplementary Figure S6). PGC1α, activated by AMPK, acts as a transcriptional coactivator for PPARα, estrogen-related receptors (e.g., ERRγ) and nuclear respiratory factor 1 (NRF1), which promote the expression of genes in OXPHOS, FAO, TCA cycle, and mitochondrial biogenesis (Cantó and Auwerx, 2009; Dominy and Puigserver, 2013; Clark and Parikh, 2021). Among the downstream transcriptional targets of PGC1a, TFAM plays a crucial role for maintaining mitochondrial structure, transcription and replication (Campbell et al., 2012), and SIRT3 promotes SOD2 activity to reduce ROS levels in mitochondria (Chen et al., 2011). Concurrently, the genes (*Ppargc1a*, *Ppara*, *Esrrg, Tfam, Sirt3* and *Sod2*) encoding these transcriptional regulators were all down-regulated in *Pkd1* mutant kidneys, but attenuated by all treatment (Figure 4A; Supplementary Figure S5). In addition, transcript levels (microarray) of *Ppargc1a*, its upstream inducer—*Prkaa2* (encoding AMPK α2), *Tfam* and *Sirt3* inversely correlated with 2 KW/BW% in *Pkd1* mutant kidneys (Figure 4B). Conversely, there was moderate positive correlation between *Prkaa1* (encoding AMPK α1) and 2 KW/BW% (r = 0.51, *p* = 0.0002). Western blot analysis confirmed the decreased expression of PGC1α, its transcriptional partner NRF1, and their target TFAM in *Pkd1* mutant kidneys; all these changes were significantly attenuated by the treatments (Figure 4C). qRT-PCR analysis confirmed the *Ppargc1a* gene expression result from microarray analysis (Figure 4D). Consistent with defective mitochondrial biogenesis in ADPKD, qPCR analysis showed the decreased mtDNA/nDNA ratio (a biomarker of mitochondrial mass) in *Pkd1* mutant kidneys (vs. WT). Salsalate, tolvaptan or in combination significantly increased mtDNA/nDNA (vs. untreated KO); the combination treatment significantly increased mtDNA/nDNA as compared to tolvaptan treatment alone (Figure 4E). An inverse correlation between mtDNA/nDNA and 2 KW/BW% was also observed in *Pkd1* mutant kidneys (r = −0.82, *p* < 0.0001) (Figure 4F). Taken together, our results indicate defective multiple mitochondrial quality control mechanisms in *Pkd1* mutant kidneys, which may induce mitochondrial damage and dysfunction, leading to kidney injury, inflammation, and fibrosis. Transcriptional changes in metabolic pathways suggest that salsalate, tolvaptan or in combination resulted in improved oxidative metabolism by modulating multiple quality control mechanisms, including mitochondrial biogenesis, antioxidant response, and mitophagy.

## 4 Discussion

Aberrant activation/inhibition and cross talk of various signaling pathways appear to be required for ADPKD cyst growth and disease progression. Therapies targeting key points of convergence in these pathways may provide novel therapeutic strategies for ADPKD (Song et al., 2009). Drugs acting on different molecular targets in cells may exhibit additive or supra-additive responses, which can help to improve therapeutic efficiency and reduce side effects (Efferth and Koch, 2011). Tolvaptan is an effective treatment for ADPKD, but also causes polyuria (Torres et al., 2012; Torres et al., 2017). Studies have shown that some but not all compounds associated with the activation of AMPK attenuate cystic kidney disease in *Pkd1* mutant animal models (Takiar et al., 2011; Chiaravalli et al., 2016; Kipp et al., 2016; Riwanto et al., 2016; Warner et al., 2016; Leonhard et al., 2019; Lian et al., 2019; Torres et al., 2019; Pastor-Soler et al., 2022). Recently, we have shown that salsalate, an AMPK activator, was highly effective in slowing PKD progression in an adult onset *Pkd1* mouse model (Leonhard et al., 2019). We hypothesize that salsalate can act cooperatively with tolvaptan and thus, may be potentially useful to improve the therapeutic outcome. We tested the efficacy of the combination of tolvaptan and salsalate, and each drug individually in the same mouse model. The protocol in current study is very similar to our previous study with the exception of an extension of the study time (111–120 days of age vs. 111–115 days of age), in order to achieve a 25% kidney survival rate vs. 50% in previous studies, in the untreated mutant mice (Leonhard et al., 2019). With this modification, we anticipated to demonstrate therapeutic synergism in the combined treatment of salsalate and tolvaptan compared to either drug alone (i.e., survival difference). Using clinically relevant doses, we found the therapeutic effects of salsalate were similar to that of tolvaptan, and the combined treatment tended to be more effective than single drug treatment associated with improved kidney survival, and reduced 2 KW/BW%, cystic index, and BU levels.

By transcriptome analysis, we studied the mechanism of disease and consequences of treatments. The dysregulated pathways enriched in *Pkd1* mutant kidneys, but attenuated by the treatments, were similar as in our previous salsalate study (Leonhard et al., 2019), Heatmaps for expressed genes reveal an expression profile slightly closer to wild-type animals for combination treatment when compared to single treatment, and the combined treatment significantly altered more genes than the monotherapies thereby strengthening the drug efficacy data. Further analysis showed perturbation of specific genes/pathways associated with kidney injury-repair process, innate immunity, and multiple mitochondrial quality control mechanisms that may represent the key pathways underlying the mechanisms of disease, and attenuation by salsalate, tolvaptan and co-treatments. By targeting intracellular cAMP levels, tolvaptan affects cystic epithelial cell proliferation and fluid secretion (Gattone et al., 2003). Salsalate is known to be a non-selective AMPK activator with multiple cellular targets. Through phosphorylation of key proteins and transcription factors, AMPK integrates the signals from multiple metabolic pathways to regulate cellular proliferation, cell cycle, carbohydrate and lipid metabolism, mitochondrial function, autophagy, inflammation, fibrosis, and fluid secretion (Hardie, 2013; Garcia and Shaw, 2017; Herzig and Shaw, 2018; Steinberg and Carling, 2019; Song et al., 2020). In addition, *via* effects on mitochondrial uncoupling and NFκB signaling and potential other targets, salsalate/salicylate may also directly modulate several of these molecular pathways and cellular functions (Rumore and Kim, 2010; Smith et al., 2016). To find out how the signaling networks modulated by salsalate and tolvaptan interfere, a different experimental set-up and follow-up studies will be required.

Compared to tolvaptan treatment alone, salsalate treatment resulted in 84 dysregulated genes. We anticipate that 11 lipid metabolism genes specifically up-regulated by salsalate (many of them are the direct targets of PPARα), may be associated with the improvement of FAO in ADPKD. On the other hand, among genes specifically altered by tolvaptan, *Aqp2* was down-regulated by tolvaptan, consistent with its effect in blocking water reabsorption. We also found *Prlr* displayed higher mRNA levels in tolvaptan treated kidneys. Prolactin is a natriuretic hormone, its receptor *Prlr* is preferentially expressed in the female S2/3 of proximal tubule (Ransick et al., 2019), the natriuretic response is associated with inhibition of proximal tubular Na^+^, K^+^-ATPase activity (Ibarra et al., 2005). Another set of genes are the helix–loop–helix (HLH) protein family that is known to regulate cell proliferation and differentiation. Polycystin-1 and polycystin-2 have been showed to regulate the cell cycle through one member of HLH protein family Id2 (Li et al., 2005). We found increased mRNA levels of Id1-4 by tolvaptan treatment. However, the significance of increased expression of Id1-4 by tolvaptan are unknown.

## 5 Conclusion

Collectively, we demonstrated that salsalate therapeutic effect was similar to that of tolvaptan; the combined treatment tended to be more effective for renal cystic disease parameters and cellular signaling, but data were not significant compared to single treatments and more studies are required to show improved efficacy of combination treatments compared to single drug treatments. Potential beneficial effects of co-treatment are likely mediated by cooperatively attenuating kidney injury, cell proliferation, cell cycle progression, inflammation and fibrosis, and improving mitochondrial function, and cellular antioxidant response. The excellent safety profile of salsalate suggests that it is a highly promising candidate for drug repurposing and clinical testing in ADPKD. If supported by additional preclinical/clinical testing, salsalate-tolvaptan combination would represent a promising therapeutic strategy in the treatment of ADPKD.

## Data Availability

The datasets presented in this study can be found in online repositories. The names of the repository/repositories and accession number (s) can be found below: https://www.ncbi.nlm.nih.gov/geo/, GSE198340.
