# Supplementary material for: Preclinical evaluation of tolvaptan and salsalate combination therapy in a Pkd1-mouse model
Source: Front Mol Biosci. 2023 Jan 19;10:1058825. doi: 10.3389/fmolb.2023.1058825 (PMC9893022; doi:10.3389/fmolb.2023.1058825)
Supplement: Supplementary file 1 [file DataSheet1.PDF]

Supplementary Material

Supplementary Figures

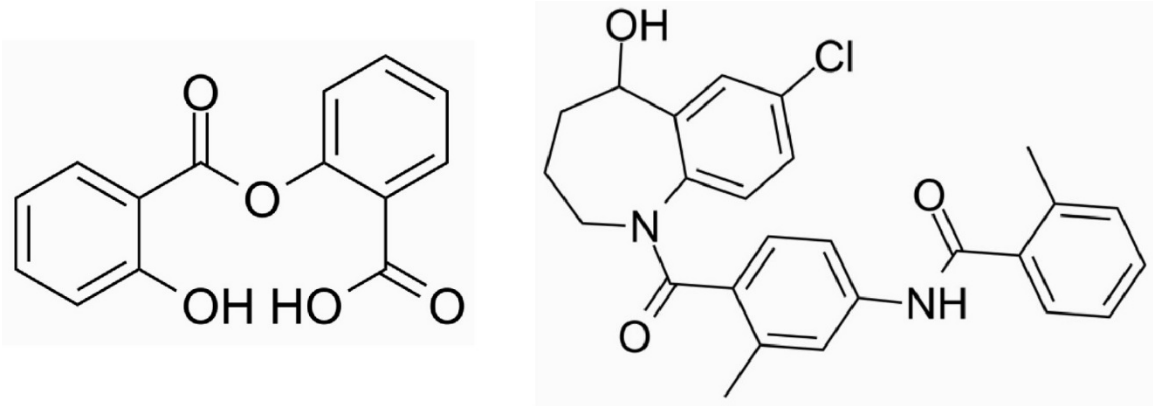

Salsalate   Tolvaptan

Supplementary Figure 1. Structures of salsalate and tolvaptan.

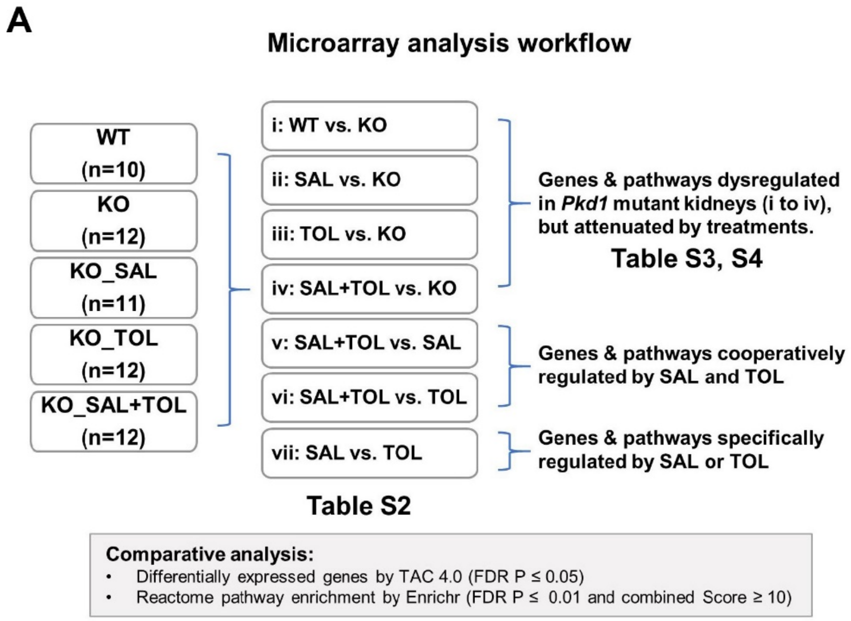

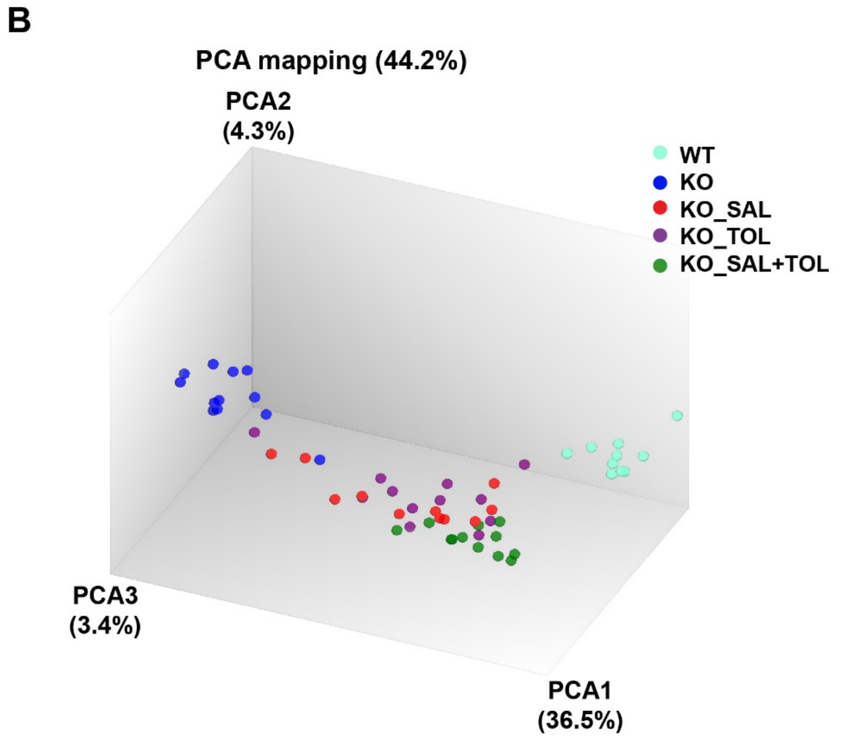

Supplementary Figure 2. **(A)** Global gene expression profiling of 57 kidney samples which were selected around the median of the 2KW/BW% from each group. Comparative analysis of WT, SAL, TOL, or SAL+TOL treated kidneys vs. the untreated KO controls, combination therapy vs. monotherapy, and two monotherapies to determine differentially expressed genes, and over-represented pathways associated with PKD disease progression, and treatment effects. **(B)** Principle component analysis (PCA) of normalized expression levels of 5,000 independent transcripts. The first three principal components accounted for 44.2% of the explained variance and clustered apart samples from different genotypes, untreated and treated groups.

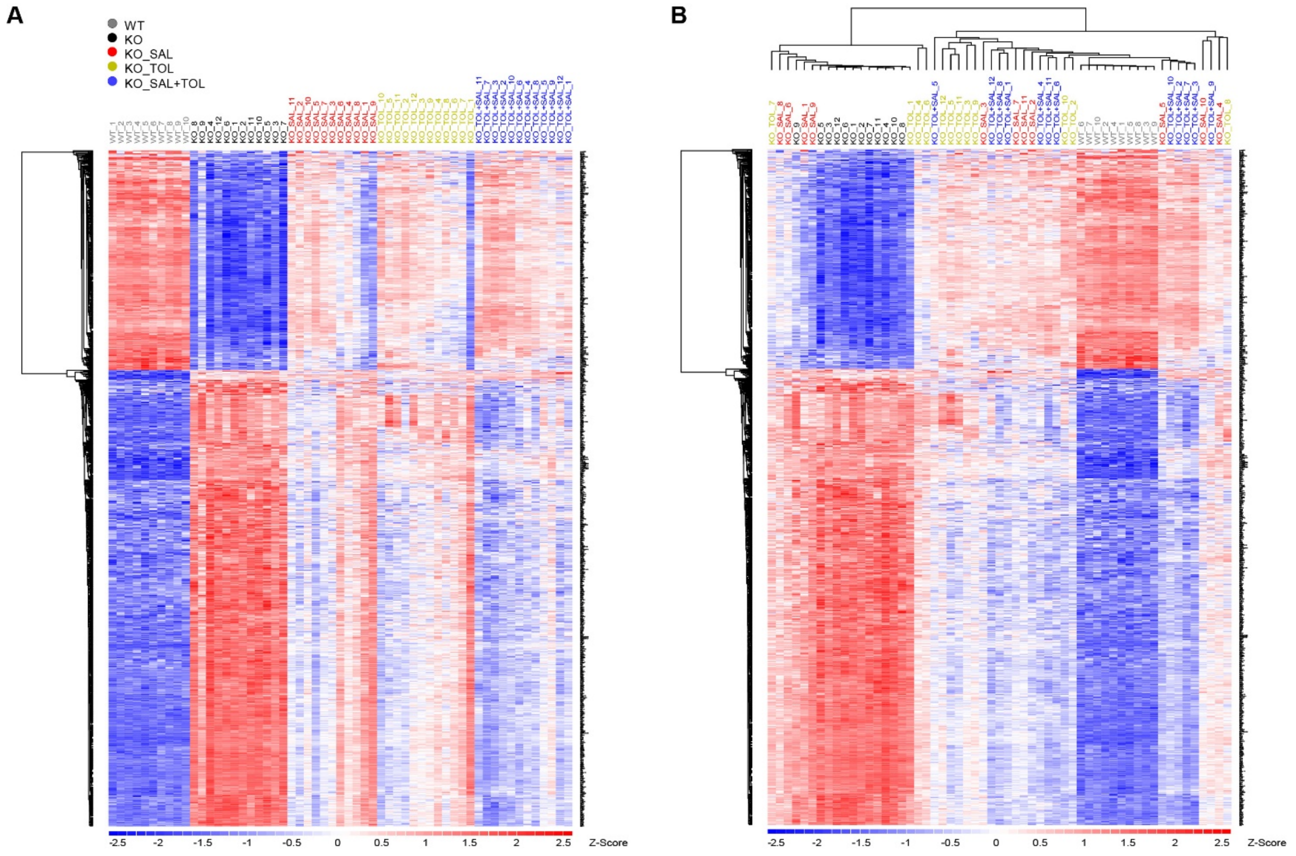

Supplementary Figure 3. Hierarchical clustering using top 1000 differentially expressed genes between KO (n=12) and WT (n=10) kidneys showing the SAL (n=11), TOL (n=12), and SAL+TOL (n=12) treatments attenuated gene expression changes in *Pkd1* mutant kidneys. **(A)** Clustering genes only, and *Pkd1* mutant kidneys were ordered by 2KW/BW% in each group. The row dendrogram shows the distance or similarity between genes. **(B)** Clustering both genes and samples. The row and column dendrograms display the distance or similarity between genes and samples, respectively. In the heatmap, each column represents an individual sample; each row represents the Z-score scaled gene expression levels across all samples. White is the mean Z-score (set to 0), red indicates greater than the mean and blue, less than the mean.

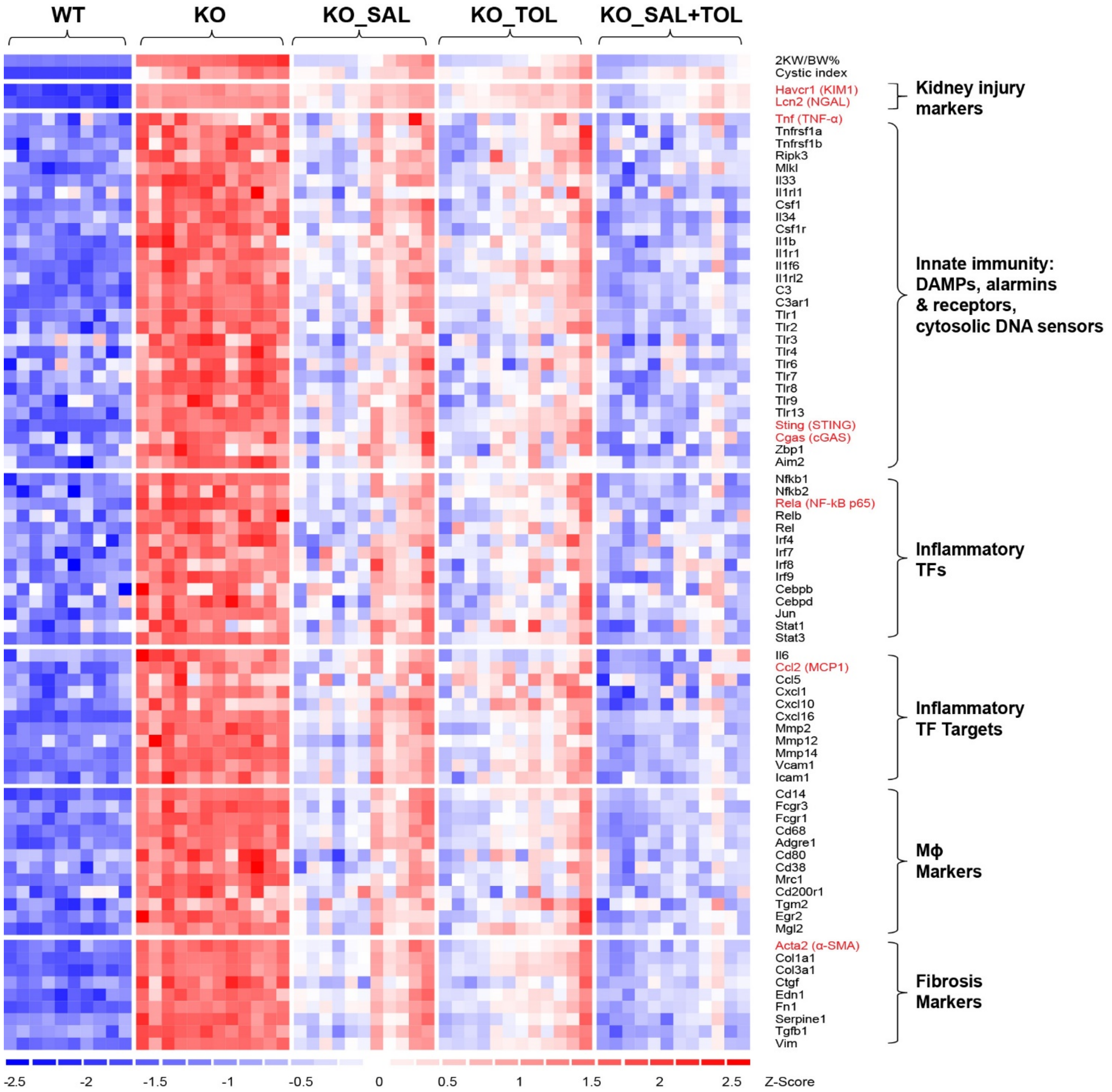

Supplementary Figure 4. The combined treatment attenuated kidney injury, inflammation, and fibrosis in *Pkd1* mutant mouse kidneys. Global gene profiling showed the increased mRNA expression of kidney injury markers, damage-associated molecular patterns (DAMPs) and their receptors, macrophages (MΦ) markers, inflammation, and fibrosis in *Pkd1* mutant kidneys; these changes were attenuated by treatments (all changes shown were identified using an FDR adjusted p-value ≤ 0.05). In the heatmap, each column represents an individual sample; each row represents the Z-score scaled gene expression levels across all samples. White is the mean Z-score (set to 0), red indicates greater than the mean and blue, less than the mean. *Pkd1* mutant kidneys in the heatmap were ordered by 2KW/BW% in each group. Genes in red were used for linear regression analysis, validated by Western blot or qRT-PCR.

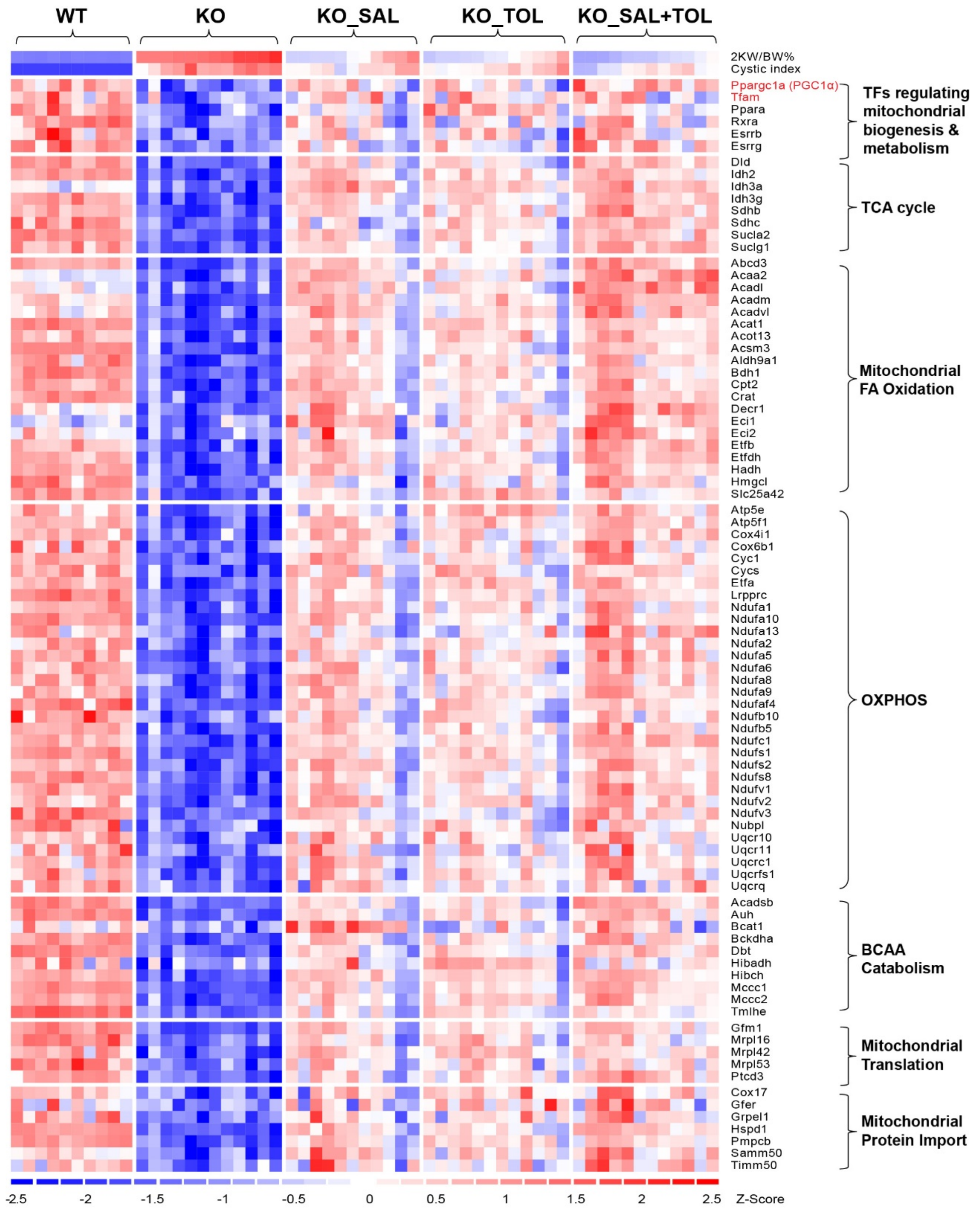

Supplementary Figure 5. The combined treatment improved expression of components of mitochondrial metabolic pathways in *Pkd1* mutant kidneys. Global gene profiling showed the changes consistent with a generalized defective mitochondrial metabolism in *Pkd1* mutant kidneys; these changes were attenuated by treatments (all changes shown were identified using an FDR adjusted p-value ≤ 0.05). In the heatmap, each column represents an individual sample; each row represents the Z-score scaled gene expression levels across all samples. White is the mean Z-score (set to 0), red indicates greater than the mean and blue, less than the mean. *Pkd1* mutant kidneys in the heatmap were ordered by 2KW/BW% in each group. Genes in red were used for linear regression analysis, validated by Western blot or qRT-PCR.

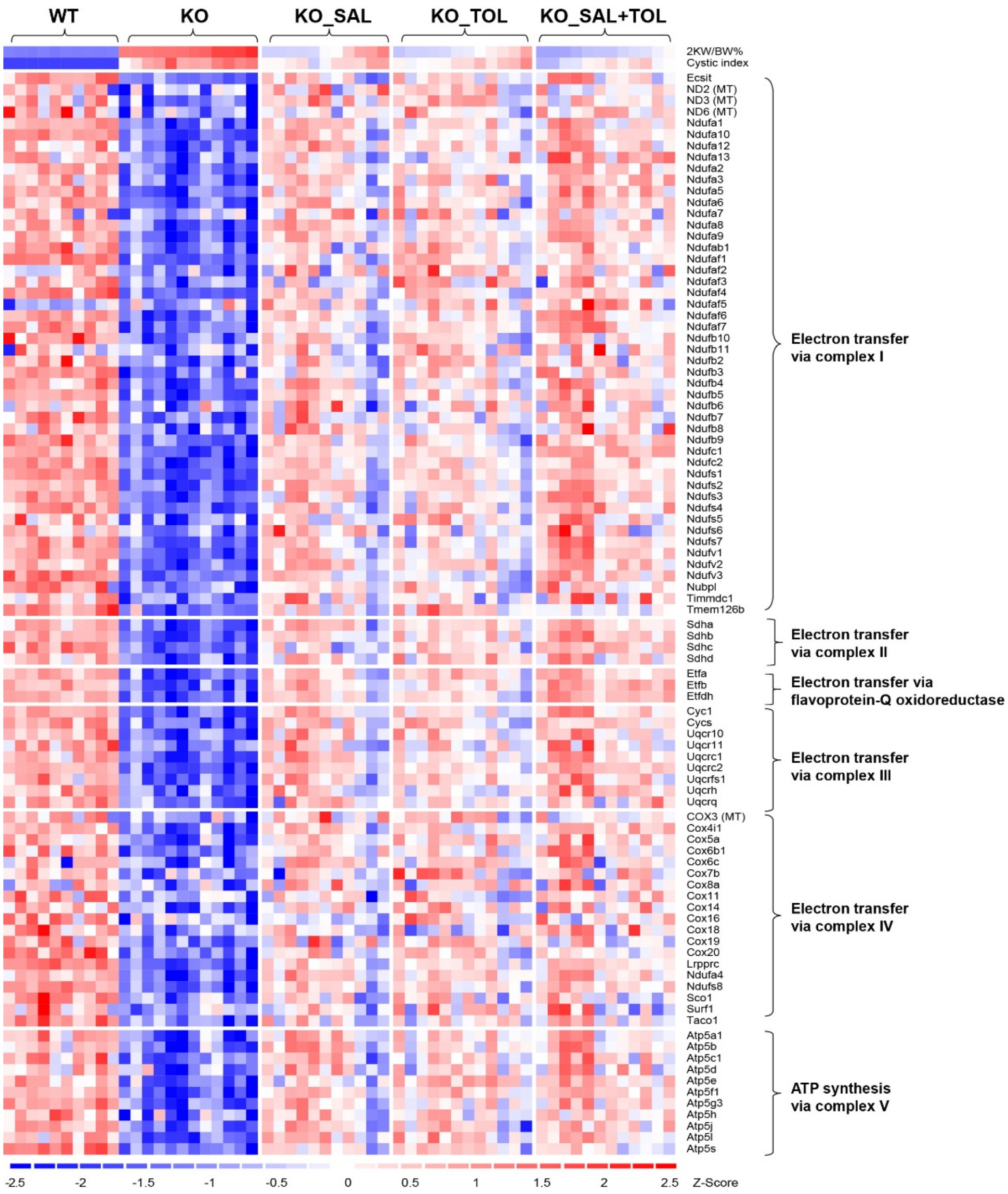

Supplementary Figure 6. The combined treatment improved expression of components of the OXPHOS complexes in *Pkd1* mutant kidneys. Global gene profiling showed the changes consistent with a generalized depressed OXPHOS in *Pkd1* mutant kidneys; these changes were attenuated by treatments (all changes shown were identified using an FDR adjusted p-value ≤ 0.05). In the heatmap, each column represents an individual sample; each row represents the Z-score scaled gene expression levels across all samples. White is the mean Z-score (set to 0), red indicates greater than the mean and blue, less than the mean. *Pkd1* mutant kidneys in the heatmap were ordered by 2KW/BW% in each group.

The OXPHOS system consists of five multiprotein complexes, the individual subunits of which are encoded either by the mitochondrial or by the nuclear genome. The electron transport chain comprises some 80 proteins organized in four enzymatic complexes (I-IV). Complex I is the largest enzyme complex in the electron transport chain, containing 45 subunits. Seven subunits (ND1-6, ND4L) are encoded by mtDNA, the remainder encoded in the nucleus. Electron transfer via flavoprotein-Q

oxidoreductase represents a major source of reducing power for the electron transport chain from FAO and amino acid degradation, is also part of the electron transport chain. Complex V generates ATP but has no electron transfer activity. There are 104 respiratory electron transport and ATP synthesis genes found in mouse. Compared to WT kidneys, 94 out of 104 of these genes were downregulated in mutant kidneys. Salsalate, tolvaptan alone or in combination significantly improved the OXPHOS system associated with an induction of mRNA expression of these genes. Mouse mtDNA genome encodes 37 genes; 13 of them encode core protein subunits of complexes I, III, IV, and V. Among these genes, Nd2/3/6, and Cox3 displayed differential expression.
